# Measles Outbreak — California, December 2014–February 2015

**Published:** 2015-02-20

**Authors:** Jennifer Zipprich, Kathleen Winter, Jill Hacker, Dongxiang Xia, James Watt, Kathleen Harriman

**Affiliations:** 1 California Department of Public Health

On January 5, 2015, the California Department of Public Health (CDPH) was notified about a suspected measles case. The patient was a hospitalized, unvaccinated child, aged 11 years with rash onset on December 28. The only notable travel history during the exposure period was a visit to one of two adjacent Disney theme parks located in Orange County, California. On the same day, CDPH received reports of four additional suspected measles cases in California residents and two in Utah residents, all of whom reported visiting one or both Disney theme parks during December 17–20. By January 7, seven California measles cases had been confirmed, and CDPH issued a press release and an Epidemic Information Exchange (Epi-X) notification to other states regarding this outbreak. Measles transmission is ongoing (Figure).

As of February 11, a total of 125 measles cases with rash occurring during December 28, 2014–February 8, 2015, had been confirmed in U.S. residents connected with this outbreak. Of these, 110 patients were California residents. Thirty-nine (35%) of the California patients visited one or both of the two Disney theme parks during December 17–20, where they are thought to have been exposed to measles, 37 have an unknown exposure source (34%), and 34 (31%) are secondary cases. Among the 34 secondary cases, 26 were household or close contacts, and eight were exposed in a community setting. Five (5%) of the California patients reported being in one or both of the two Disney theme parks during their exposure period outside of December 17–20, but their source of infection is unknown. In addition, 15 cases linked to the two Disney theme parks have been reported in seven other states: Arizona (seven), Colorado (one), Nebraska (one), Oregon (one), Utah (three), and Washington (two), as well as linked cases reported in two neighboring countries, Mexico (one) and Canada (10).

Among the 110 California patients, 49 (45%) were unvaccinated; five (5%) had 1 dose of measles-containing vaccine, seven (6%) had 2 doses, one (1%) had 3 doses, 47 (43%) had unknown or undocumented vaccination status, and one (1%) had immunoglobulin G seropositivity documented, which indicates prior vaccination or measles infection at an undetermined time. Twelve of the unvaccinated patients were infants too young to be vaccinated. Among the 37 remaining vaccine-eligible patients, 28 (67%) were intentionally unvaccinated because of personal beliefs, and one was on an alternative plan for vaccination. Among the 28 intentionally unvaccinated patients, 18 were children (aged <18 years), and 10 were adults. Patients range in age from 6 weeks to 70 years; the median age is 22 years. Among the 84 patients with known hospitalization status, 17 (20%) were hospitalized.

The source of the initial Disney theme park exposure has not been identified. Specimens from 30 California patients were genotyped; all were measles genotype B3, which has caused a large outbreak recently in the Philippines, but has also been detected in at least 14 countries and at least six U.S. states in the last 6 months (1).

Annual attendance at Disney theme parks in California is estimated at 24 million (2), including many international visitors from countries where measles is endemic. The December holiday season coincides with the exposure period of interest. Since 2011, six confirmed measles cases have been reported to CDPH in persons whose notable exposure was to large theme parks that attract international tourists. International travel to countries where measles is endemic is a well-known risk factor for measles, and measles importations continue to occur in the United States; the number of measles cases reported to CDC is updated weekly at http://www.cdc.gov/measles/cases-outbreaks.html. However, U.S. residents also can be exposed to measles in the United States at venues with large numbers of international visitors, such as other tourist attractions and airports. This outbreak illustrates the continued importance of ensuring high measles vaccination coverage in the United States.

## Figures and Tables

**FIGURE:**
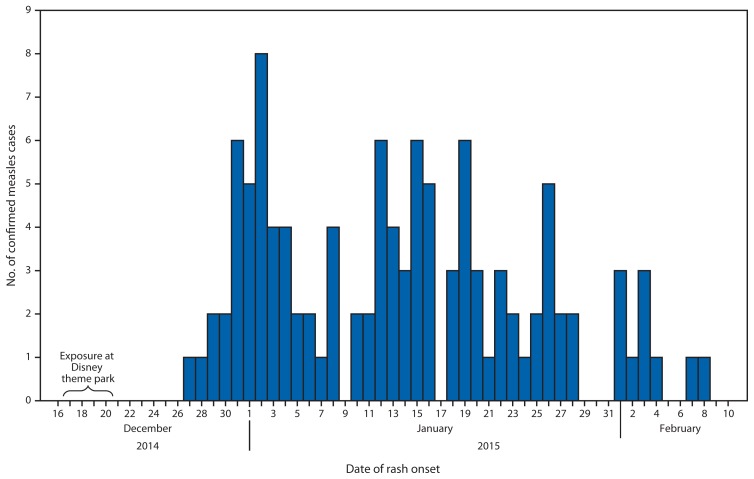
Number of confirmed measles cases (N = 110),* by date of rash onset — California, December 2014–February 2015 *Reported to the California Department of Public Health as of February 11, 2015.
