# Errata

**Published:** 2015-02-27

## Vol. 64, No. 6

In the report, “Measles Outbreak — California, December 2014–February 2015,” two errors occurred.

In the second paragraph, the sixth sentence should read, “In addition, 15 cases linked to the two Disney theme parks have been reported in seven other states: Arizona (seven), Colorado (one), Nebraska (one), Oregon (one), **Texas (one),** Utah (three), and Washington (two), as well as linked cases reported in two neighboring countries, Mexico (one) and Canada (10).”

In the third paragraph, the third sentence should read, “Among the 37 remaining vaccine-eligible patients, 28 (**76%**) were intentionally unvaccinated because of personal beliefs, and one was on an alternative plan for vaccination.”

